# Association between nutritional‑inflammatory status and diabetic retinopathy in adults with diabetes: insights from a US population‑based survey and a Chinese hospital‑based clinical cohort

**DOI:** 10.3389/fendo.2026.1883581

**Published:** 2026-08-06

**Authors:** Jian Deng, Liduo Pan, Qi Zhang, Meng Wang, Shijun Luo, Yonghao Li

**Affiliations:** 1 Department of Ophthalmology, Aier Eye Hospital, Jinan University, Guangzhou, Guangdong, China; 2 Department of Ophthalmology, Shenzhen Eye Hospital, Shenzhen Eye Medical Center, Southern Medical University, Shenzhen, Guangdong, China

**Keywords:** diabetic retinopathy, inflammation, NHANES, nutrition, clinical cohort, risk stratification

## Abstract

**Background:**

Diabetic retinopathy (DR) is the predominant cause of vision loss in working-age adults. The glucocentric model cannot fully account for its pathogenesis, and emerging evidence highlights the significant roles of nutritional status and inflammation. However, the precise value of composite nutritional-inflammatory-related indices in DR has yet to be systematically evaluated in diverse ethnic populations.

**Methods:**

This dual-cohort study analyzed data from 3,715 adults with diabetes in NHANES (2005–2018) and a hospital-based clinical cohort of 252 type 2 diabetes patients from a tertiary eye center. We systematically examined associations between DR and the prognostic nutritional index (PNI), geriatric nutritional risk index (GNRI), albumin-bilirubin (ALBI) score, and neutrophil percentage-to-albumin ratio (NPAR) using weighted multivariable logistic regression, restricted cubic splines (RCS), and subgroup analyses. Threshold effect analysis identified critical inflection points for nonlinear associations. Three sensitivity analyses were conducted.

**Results:**

In the NHANES cohort after multivariable adjustment, the highest versus lowest tertiles of PNI (OR = 0.57; 95% CI: 0.41–0.80) and GNRI (OR = 0.56; 95% CI: 0.41–0.76) were associated with reduced DR risk, whereas the highest tertiles of ALBI (OR = 1.58; 95% CI: 1.18–2.19) and NPAR (OR = 1.37; 95% CI: 1.02–1.83) conferred elevated risk, with all relationships demonstrating significant dose–response trends. These associations were corroborated, with even stronger effect sizes, in the hospital-based clinical cohort, where PNI and GNRI declined progressively with increasing DR severity, while ALBI and NPAR rose incrementally. RCS confirmed nonlinear associations between PNI/GNRI and DR, and threshold effect analyses identified cohort-specific inflection points (NHANES: PNI = 56.00, GNRI = 98.27; clinical cohort: PNI = 42.85, GNRI = 90.39). Subgroup analyses showed stronger protective effects of PNI among overweight/obese individuals and stronger risk associations for ALBI in women. Sensitivity analyses confirmed the robustness of these findings.

**Conclusions:**

This dual-cohort study provides robust and cross-ethnic evidence that PNI and GNRI are inversely linked to DR, whereas ALBI and NPAR show positive associations, and the hospital-based clinical cohort further links these indices to DR severity. These findings highlight the utility of these accessible nutritional-inflammatory-related indices as pragmatic tools for DR risk stratification and early warning.

## 1 Introduction

Diabetes mellitus represents a significant global public health issue, with the International Diabetes Federation (IDF) forecasting that the global prevalence will rise to 783 million individuals by 2045 (1). Diabetic retinopathy (DR) is one of the most prevalent complications of type 2 diabetes and is the leading cause of vision impairment and blindness in the working-age population (2). The intersection of increasing diabetes prevalence and an ageing population has led to a continual rise in DR caseloads, which is significantly impairing patients’ quality of life and exerting a notable socioeconomic burden on healthcare systems (3). Intensive glycaemic control has been clearly demonstrated to lower the risk of DR onset and progression by up to 50% [4], but new clinical data highlights a recurring paradox: 20% of patients develop retinopathy even after reaching ideal glycaemic objectives (4–6). This disconnect strongly indicates that a purely “glucocentric” paradigm is insufficient to fully elucidate the complex pathogenesis of DR (5, 6). Consequently, the identification of novel biomarkers is imperative, not merely to facilitate early prevention and targeted intervention, but also to serve as a critical adjunct to conventional glycemic management strategies.

The inflammatory status is closely linked to the onset and advancement of DR. Elevated levels of inflammatory markers such as IL-6, TNF-α, and MCP-1 have been observed in the aqueous humor of patients with DR (7). Meanwhile, systemic inflammatory indicators derived from peripheral blood, including the lymphocyte-to-monocyte ratio (LMR), neutrophil-to-lymphocyte ratio (NLR), and systemic immune-inflammation index (SII), can be used to predict DR occurrence and guide treatment decisions (8, 9). The nutritional status is pivotal in the management of DR (10). Serum albumin, a crucial marker of nutritional status, might prevent the progression of DR through its antioxidant and anti-inflammatory attributes (11). Given that the pathogenesis of DR involves interactions among multiple pathological processes, including metabolism, inflammation, and nutrition, the use of a single type of biomarker has inherent limitations in comprehensively assessing disease risk. Consequently, incorporating nutritional and inflammatory information into a composite indicator may enhance the predictive value for DR risk.

The significance of nutritional-inflammatory-related indices in predicting disease prognosis has attracted academic recognition in recent years. Indicators such as the prognostic nutritional index (PNI), neutrophil percentage-to-albumin ratio (NPAR), geriatric nutritional risk index (GNRI), and albumin-bilirubin score (ALBI) can be readily derived from routine clinical laboratory data. Recent studies have validated their substantial utility in forecasting mortality and complications across diverse cardiovascular diseases, cancers, and diabetes mellitus (12–16). Nevertheless, although these indicators accurately reflect the body’s inflammatory load and nutritional status, their clinical utility in patients with DR remains underexplored and warrants further investigation across diverse populations.

Therefore, we performed a cross-sectional study utilizing the National Health and Nutrition Examination Survey (NHANES) database and employed a hospital-based clinical cohort from a tertiary eye center to systematically evaluate the relationship between four nutritional-inflammatory-related (PNI, ALBI, NPAR, and GNRI) and DR. This study enhances comprehension of the interplay between nutrition and inflammation in the pathogenesis of DR, addressing the explanatory limitations of the conventional “glucose-centric” model, and provides novel insights for early prevention and intervention strategies for DR.

## 2 Methods

### 2.1 NHANES cohort

**Figure 1:**
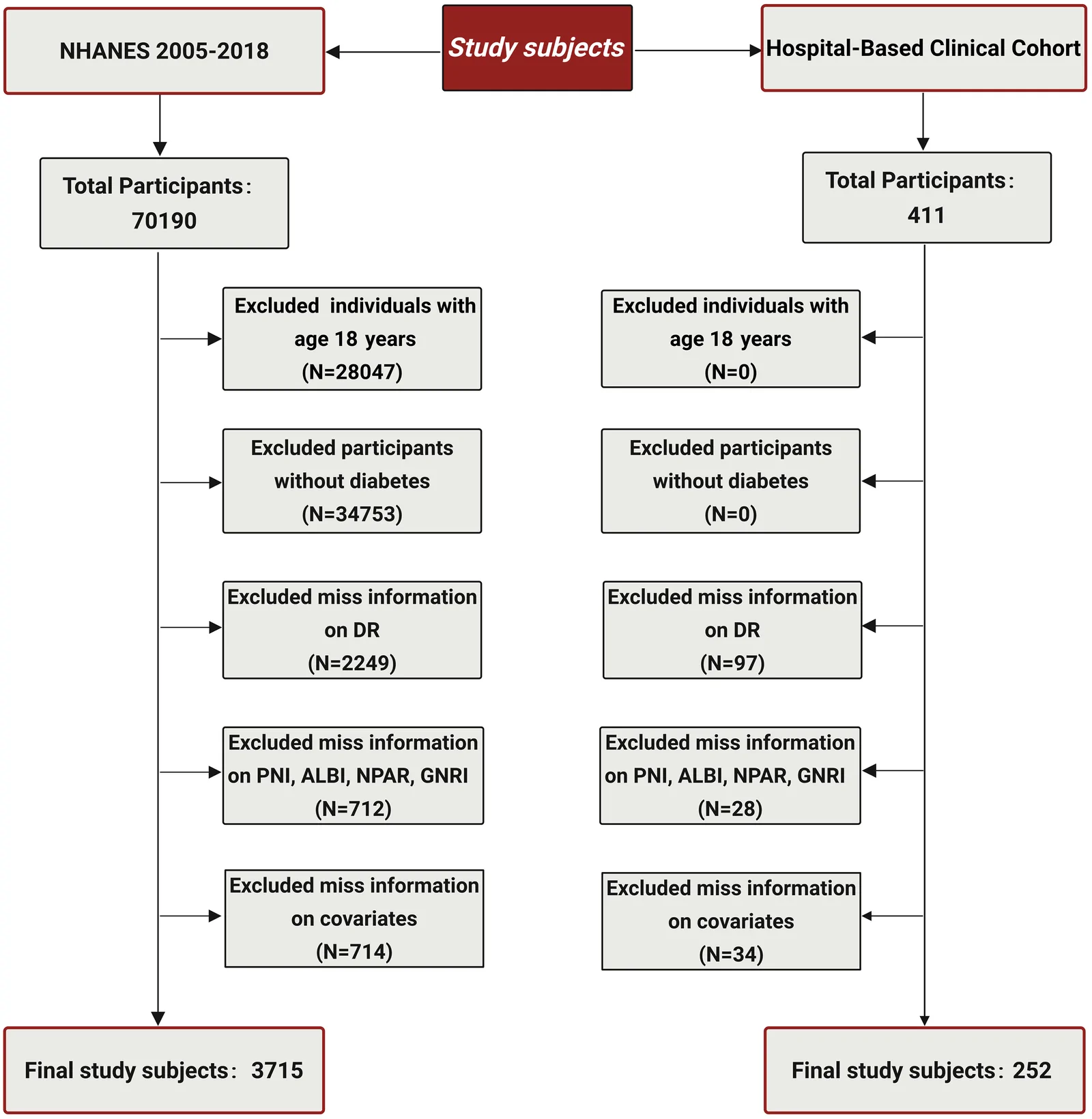
An overview of the study design. NHANES, National Health and Nutrition Examination Survey; DR, diabetic retinopathy; PNI, prognostic nutritional index; NPAR, neutrophil percentage to albumin ratio; ALBI, albumin-bilirubin; GNRI, geriatric nutritional risk index.

The NHANES is a nationally representative, multistage, probability-stratified survey conducted by the National Center for Health Statistics (NCHS) to systematically collect health, nutrition, and lifestyle data from the non-institutionalized U.S. population. This study included data from the NHANES 2005–2018 cycles. All NHANES protocols were approved by the NCHS Ethics Review Board, and written informed consent was obtained from all participants. A total of 70,190 participants were initially enrolled. Participants were sequentially excluded based on the following criteria: age <18 years (n=28,047), missing diabetes diagnosis data (n=34,753), missing DR data (n=2,249), incomplete inflammatory or nutritional indicator data (n=712), and missing data for other key covariates (n=714). The final analysis included 3,715 patients with diabetes. The detailed participant selection process is illustrated in **Figure 1**. This study adhered to the Strengthening the Reporting of Observational Studies in Epidemiology (STROBE) reporting guidelines (**Supplementary Table 1**).

### 2.2 Hospital-based clinical cohort

To assess the generalizability of the findings from the NHANES dataset, we employed an independent clinical cohort for real-world supplementary analysis. This cohort comprised 411 patients with type 2 diabetes mellitus who attended Guangzhou Aier Eye Hospital, affiliated with Jinan University, from June 2024 to December 2025. The diagnosis of diabetes followed the American Diabetes Association criteria utilized for the NHANES cohort (17). This study received approval from the Ethics Committee of Guangzhou Aier Eye Hospital (approval No. GZAIER-2025-IRB-46) and adhered strictly to the principles of the Declaration of Helsinki. The exclusion criteria for the cohort included (1): type 1 diabetes (2); retinal disorders unrelated to diabetes (e.g., hypertensive retinopathy, retinal vein occlusion, and macular edema from other causes) (3); severe liver conditions such as cirrhosis or hepatocellular carcinoma (4); end-stage renal disease or patients on dialysis (5); active infections, malignancy, autoimmune disease, congestive heart failure (NYHA class III–IV), or gestational diabetes; and (6) significant trauma or surgical procedures within the preceding six months. On this basis, cases with missing data were further excluded: missing DR diagnosis data (n=97), incomplete inflammatory or nutritional indicator data (n=28), and missing data for other key covariates (n=34). A total of 252 participants with type 2 diabetes were included in the supplementary clinical cohort analysis. The detailed participant selection process is shown in **Figure 1**.

### 2.3 Exposure variables and outcome definition

Participants were diagnosed with diabetes based on any of the following criteria (1): fasting glucose ≥7.0 mmol/L (2); random glucose ≥11.1 mmol/L (3); 2-hour glucose ≥11.1 mmol/L during OGTT (4); HbA1c ≥6.5% (5); use of glucose-lowering medications or insulin; or (6) physician-diagnosed diabetes. These criteria align with American Diabetes Association guidelines (17). The primary exposure variables in this study included PNI, NPAR, GNRI, and ALBI. The precise computation methods for these inflammatory-nutritional indices were as follows:

1. PNI = 10 × albumin (g/dL) + 0.005 × absolute lymphocyte count (10^3^ cells/μL) (18);
2. NPAR = (neutrophil percentage × 100)/albumin (g/dL) (19);
3. GNRI = 14.89 × albumin (g/dL) + (41.7 × actual BMI/ideal BMI), with the ideal BMI defined as 22 kg/m2 (20);
4. ALBI = [log10[total bilirubin (µmol/L)] × 0.66] − [albumin (g/L) × 0.085] (21).

DR status was the primary outcome variable. In the NHANES cohort, DR was assessed using the questionnaire item: “Has diabetes affected your eyes or have you ever had retinopathy?” Participants answering “yes” were defined as DR-positive. This self-reported approach has been widely used in previous NHANES-based studies on diabetic complications, offering both feasibility and reasonable reliability (22). In the hospital-based clinical cohort, all patients underwent comprehensive fundus examinations, including dilated ophthalmoscopy, color fundus photography, and optical coherence tomography (OCT). DR was diagnosed and graded by two retinal specialists using the International Clinical Diabetic Retinopathy (ICDR) severity scale as no DR, non-proliferative diabetic retinopathy (NPDR), or proliferative diabetic retinopathy (PDR) (23).

### 2.4 Covariates

Covariates were selected based on known confounders from previous studies and clinical practice to adjust for potential confounding. In the NHANES analysis, covariates included demographic variables (age, sex, race, marital status, education level), lifestyle factors (smoking status, alcohol use, BMI), laboratory indicators (HbA1c, eGFR), and medical history (hypertension, hyperlipidemia). Hypertension was defined as mean systolic blood pressure ≥140 mmHg or diastolic blood pressure ≥90 mmHg (mean of three measurements), antihypertensive medication use, or physician diagnosis. Hyperlipidemia was defined as any of the following: triglycerides ≥150 mg/dL, total cholesterol ≥200 mg/dL, LDL-C ≥130 mg/dL, HDL-C ≤40 mg/dL in men or ≤50 mg/dL in women, or receipt of lipid-lowering therapy. Race was categorized as Mexican American, other Hispanic, non-Hispanic White, non-Hispanic Black, or other race. Education level was classified as below high school, high school, or above high school. Smoking status was categorized as never, former, or current. Alcohol use was classified as yes or no based on questionnaire. BMI was defined according to World Health Organization guidelines as: normal or underweight (<25.0 kg/m²), overweight (25.0–30.0 kg/m²), and obese (≥30.0 kg/m²). In the clinical cohort, race was not included as a covariate due to the homogeneous racial composition of participants, whereas all other covariates were defined similarly to those in the NHANES analysis.

### 2.5 Two-piecewise linear regression analysis

To identify key inflection points in the nonlinear association between nutritional-inflammatory-related indices and DR, we used a two-piece linear regression model. This model examines slope changes at specified inflection points to identify possible threshold effects, with the inflection point established through likelihood ratio testing to optimize model fit. This approach helps clarify whether specific cutoff values of these indicators significantly alter their association with DR.

### 2.6 Statistical analysis

Given the complex, multistage, stratified sampling design of the NHANES cohort, all analyses incorporated sample weights. Continuous variables were presented as mean (standard deviation, SD), and categorical variables as frequency (percentage). For between-group comparisons, statistical tests were chosen based on data distribution: one-way ANOVA or Kruskal–Wallis for continuous variables, and chi-square for categorical variables. To investigate the correlation between nutritional-inflammatory-related indices and the risk of DR, each index was analyzed as both a continuous variable and categorized into tertiles. Results were presented as odds ratios (ORs) with 95% confidence intervals (CIs), and the P for trend was calculated. Three multivariate logistic regression models were constructed. Model 1 was unadjusted. Model 2 was adjusted for sociodemographic covariates, including age, sex, race, marital status, and educational attainment. Model 3 further adjusted for lifestyle factors, medical history, and clinical indicators, encompassing smoking status, alcohol use, body mass index (BMI), hemoglobin A1c (HbA1c), estimated glomerular filtration rate (eGFR), hypertension, and hyperlipidemia. In Model 3, covariates were chosen based on prior research, clinical significance, and variance inflation factor analysis to prevent multicollinearity. Race was excluded from the clinical cohort due to homogeneous composition. We calculated the Spearman correlation coefficients among the four indices in the NHANES cohort and presented them in **Supplementary Figure 1**, with coefficients ranging from 0.49 to 0.87. Notably, none of these indices were entered into the same regression model simultaneously to eliminate bias in effect estimates caused by multicollinearity. All covariates were adjusted simultaneously within each model, with variance inflation factor values all below 10.

To explore potential nonlinear relationships between nutritional-inflammatory-related indices and DR, restricted cubic spline (RCS) analysis was performed with adjustment for all covariates in Model 3.

We also conducted subgroup analyses to assess whether the association between nutritional-inflammatory-related indices and DR varied across population characteristics. Stratification variables included age (18–45, 45–60, ≥60 years), sex (male/female), education level (below high school, high school, above high school), BMI (normal/underweight, overweight, obese), smoking (never, former, current), alcohol use (yes/no), hypertension (yes/no), and hyperlipidemia (yes/no). We tested interactions by adding the product term of the exposure variable and each stratification variable to the model.

Finally, to assess the robustness of the findings from the NHANES cohort, we performed several sensitivity analyses, including using unweighted data, excluding extreme values, and adjusting for additional covariates. All statistical analyses were conducted using R software (version 4.4.3). A two-sided *P*-value < 0.05 was considered statistically significant.

## 3 Results

### 3.1 Baseline characteristics

The final analysis included two independent cohorts. **Table 1** presents the baseline characteristics of the NHANES cohort, which comprised 3,715 eligible participants from 2005 to 2018. The mean age was 59.69 ± 13.25 years, with 53.0% males. DR was present in 773 participants (20.80%). No significant between-group differences were observed in age, sex, race, marital status, smoking, BMI, or hyperlipidemia prevalence. Compared with non-DR diabetic patients, those with DR had a higher proportion of education below high school, lower PIR, lower rate of alcohol use, and higher prevalence of hypertension, along with lower eGFR and higher HbA1c levels. Regarding nutritional-inflammatory-related indices, DR patients exhibited higher ALBI and NPAR, but lower PNI and GNRI.

**Table 1:** Baseline characteristics of participants in NHANES cohort.

Variable	Total (n=3715)	Non-DR (n=2942)	DR (n=773)	P value
Age, y	59.69 ± 13.25	59.63 ± 13.34	59.99 ± 12.84	0.974
Gender, n (%)				0.575
Male	1,962 (53%)	1,531 (52%)	431 (56%)	
Female	1,753 (47%)	1,4 11 (48%)	342 (44%)	
Race, n (%)				0.194
Mexican American	1,380 (37%)	1,116 (38%)	264 (34%)	
Other Hispanic	952 (26%)	740 (25%)	212 (27%)	
Non-Hispanic White	658 (18%)	533 (18%)	125 (16%)	
Non-Hispanic Black	374 (10%)	287 (9.8%)	87 (11%)	
Other Race	351 (9.4%)	266 (9.0%)	85 (11%)	
Marital status, n (%)				0.574
Married/living with partner	2,228 (60%)	1,773 (60%)	455 (59%)	
Widowed/separated/divorced	1,171 (32%)	915 (31%)	256 (33%)	
Never married	316 (8.5%)	254 (8.6%)	62 (8.0%)	
Education level, n (%)				0.012
Less than high school	625 (17%)	480 (16%)	145 (19%)	
High school	1,486 (40%)	1,179 (40%)	307 (40%)	
Higher than high school	1,604 (43%)	1,283 (44%)	321 (42%)	
**PIR**	2.77 ± 1.60	2.82 ± 1.59	2.52 ± 1.62	**<0.001**
Smoking status, n (%)				0.619
Never	1,828 (49%)	1,452 (49%)	376 (49%)	
Former	1,314 (35%)	1,025 (35%)	289 (37%)	
Now	573 (15%)	465 (16%)	108 (14%)	
Drinking status, n (%)				0.016
No	1,210 (33%)	947 (32%)	263 (34%)	
Yes	2,505 (67%)	1,995 (68%)	510 (66%)	
Hypertension, n (%)				0.002
No	921 (25%)	772 (26%)	149 (19%)	
Yes	2,794 (75%)	2,170 (74%)	624 (81%)	
Hyperlipidemia, n (%)				0.180
No	448 (12%)	374 (13%)	74 (9.6%)	
Yes	3,267 (88%)	2,568 (87%)	699 (90%)	
BMI, kg/m2	33.20 ± 7.54	33.14 ± 7.43	33.47 ± 8.01	0.755
eGFR, mL/min/1.73m²	81.81 ± 25.15	83.05 ± 23.79	76.49 ± 29.77	**0.001**
HbA1c, %	7.30 ± 1.69	7.22 ± 1.66	7.68 ± 1.77	**<0.001**
PNI	52.16 ± 6.69	52.45 ± 6.85	50.90 ± 5.77	**<0.001**
NPAR	14.72 ± 2.75	14.61 ± 2.68	15.17 ± 3.01	**<0.001**
ALBI	-2.86 ± 0.30	-2.87 ± 0.29	-2.80 ± 0.31	**<0.001**
GNRI	103.04 ± 5.11	103.30 ± 5.04	101.92 ± 5.28	**<0.001**

Continuous variables were expressed as weighted mean ± standard deviation; one-way ANOVA was used to compare differences among the different groups. Categorical variables were expressed as weighted frequencies and percentages and compared using weighted chi-square test. The bold values indicate statistically significant differences.

DR, diabetic retinopathy; PIR, poverty income ratio; BMI, body mass index; eGFR, estimated glomerular filtration rate; HbA1c, glycosylated hemoglobin A1c; PNI, prognostic nutritional index; NPAR, neutrophil percentage to albumin ratio; ALBI, albumin-bilirubin; GNRI, geriatric nutritional risk index.

The hospital-based clinical cohort showed baseline characteristics partially similar to those of the NHANES cohort. The 252 participants had a mean age of 59.67 ± 12.74 years, with 56.0% females, and 93 (36.9%) had DR. Compared with non-DR patients, those with DR were older, had a lower education level, higher prevalence of hypertension, lower eGFR, and higher HbA1c. Regarding nutritional-inflammatory-related indices, DR patients had higher ALBI and NPAR but lower PNI and GNRI. Of note, the older age and higher DR prevalence in the clinical cohort may reflect hospital-based clustering of patients requiring diabetic complication management, who often have longer disease duration. **Table 2** details the baseline characteristics of the participants.

**Table 2:** Baseline characteristics of participants in hospital-based clinical cohort.

Variable	Total (n=252)	Non-DR (n=159)	DR (n=93)	*P* value
Age, y	59.67 ± 12.74	58.21 ± 12.92	62.15 ± 12.10	0.016
Gender, n (%)				0.517
Male	111 (44%)	73 (46%)	38 (41%)	
Female	141 (56%)	86 (54%)	55 (59%)	
Marital status, n (%)				0.223
Married/living with partner	17 (6.7%)	14 (8.8%)	3 (3.2%)	
Widowed/separated/divorced	215 (85%)	132 (83%)	83 (89%)	
Never married	20 (7.9%)	13 (8.2%)	7 (7.5%)	
Education level, n (%)				0.015
Less than high school	113 (45%)	61 (38%)	52 (56%)	
High school	61 (24%)	46 (29%)	15 (16%)	
Higher than high school	78 (31%)	52 (33%)	26 (28%)	
Smoking status, n (%)				0.986
Never	177 (70%)	112 (70%)	65 (70%)	
Former	61 (24%)	38 (24%)	23 (25%)	
Now	14 (5.6%)	9 (5.7%)	5 (5.4%)	
Drinking status, n (%)				0.985
No	218 (87%)	137 (86%)	81 (87%)	
Yes	34 (13%)	22 (14%)	12 (13%)	
Hypertension, n (%)				0.004
No	126 (50%)	91 (57%)	35 (38%)	
Yes	126 (50%)	68 (43%)	58 (62%)	
Hyperlipidemia, n (%)				0.351
No	103 (41%)	69 (43%)	34 (37%)	
Yes	149 (59%)	90 (57%)	59 (63%)	
**BMI, kg/m2**	24.47 ± 3.58	24.69 ± 3.76	24.09 ± 3.24	0.183
**eGFR, mL/min/1.73m²**	89.88 ± 24.23	95.03 ± 20.76	81.07 ± 27.17	**<0.001**
**HbA1c, %**	9.64 ± 2.82	9.24 ± 2.71	10.33 ± 2.89	**0.004**
**PNI**	51.36 ± 6.14	53.11 ± 5.58	48.38 ± 5.93	**<0.001**
**NPAR**	14.52 ± 4.21	13.61 ± 4.22	16.07 ± 3.72	**<0.001**
**ALBI**	-2.74 ± 0.34	-2.85 ± 0.31	-2.56 ± 0.30	**<0.001**
**GNRI**	101.35 ± 6.25	103.47 ± 5.59	97.71 ± 5.64	**<0.001**

Continuous variables were expressed as weighted mean ± standard deviation; one-way ANOVA was used to compare differences among the different groups. Categorical variables were expressed as weighted frequencies and percentages and compared using weighted chi-square test. The bold values indicate statistically significant differences.

DR, diabetic retinopathy; BMI, body mass index; eGFR, estimated glomerular filtration rate; HbA1c, glycosylated hemoglobin A1c; PNI, prognostic nutritional index; NPAR, neutrophil percentage to albumin ratio; ALBI, albumin-bilirubin; GNRI, geriatric nutritional risk index.

### 3.2 Association between nutritional-inflammatory-related indices and DR

**Figure 2:**
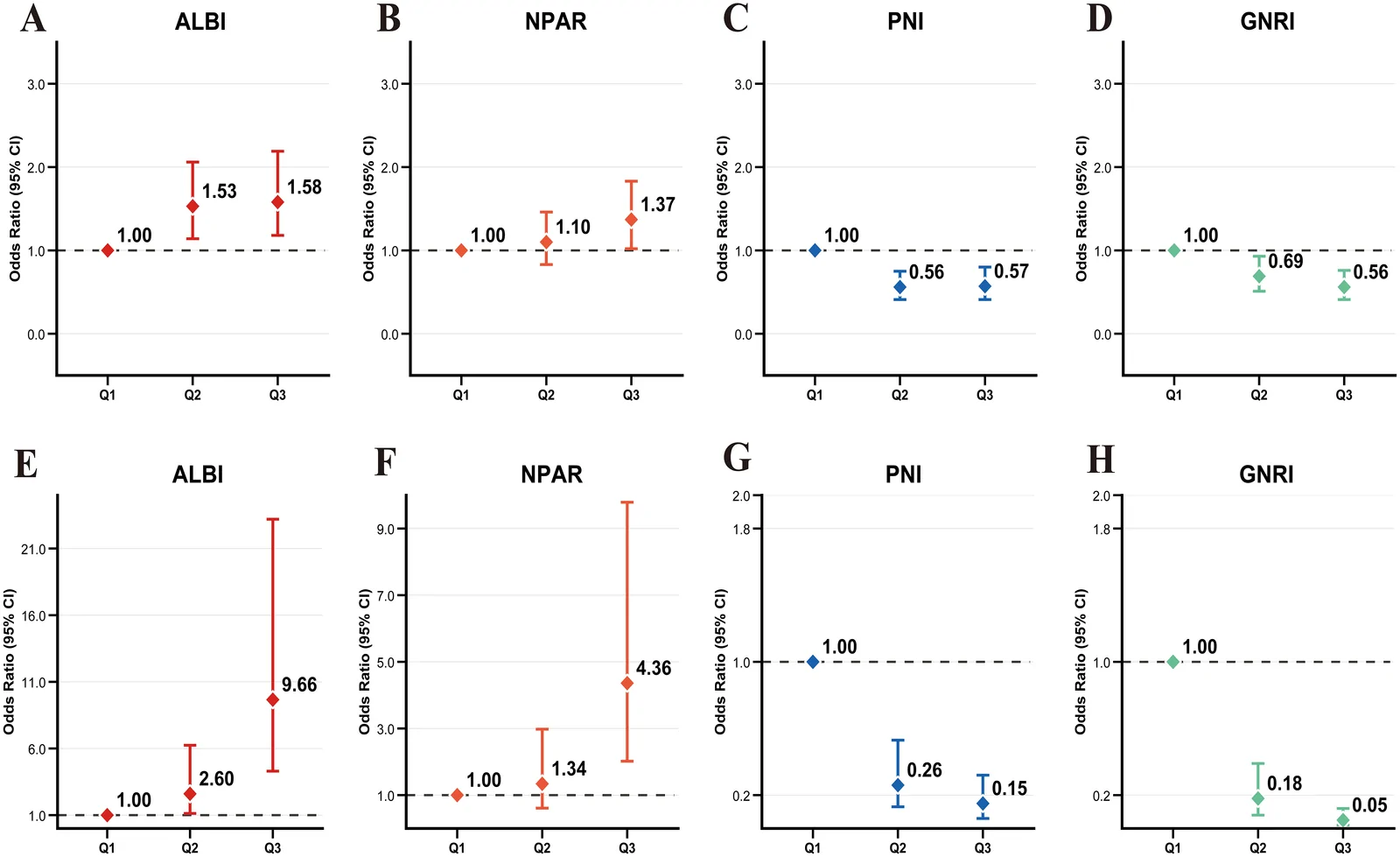
Association between nutritional-inflammatory-related indices and DR in Model 3. **(A–D)** show the risk of DR by tertile of the ALBI, NPAR, PNI, and GNRI (Model 3) in the NHANES cohort, with adjustments for age, sex, race, marital status, education level, smoking status, drinking status, DR, diabetic retinopathy BMI, HbA1c, eGFR, hypertension, and hyperlipidemia. **(E–H)** show the risk of DR by tertile of the ALBI, NPAR, PNI, and GNRI (Model 3) in the hospital-based clinical cohort, with adjustments for age, sex, marital status, education level, smoking status, drinking status, BMI, HbA1c, eGFR, hypertension, and hyperlipidemia. BMI, body mass index; eGFR, estimated glomerular filtration rate; HbA1c, glycosylated hemoglobin A1c. ALBI, albumin-bilirubin; NPAR, neutrophil percentage to albumin ratio; PNI, prognostic nutritional index; GNRI, geriatric nutritional risk index.

In weighted logistic regression analyses of the NHANES cohort, we performed separate models for each of the four nutrition-inflammation-related indices (PNI, ALBI, NPAR, and GNRI) to examine their individual associations with DR. As presented in **Table 3** and **Figure 2**. When analyzed as continuous variables in the fully adjusted model (Model 3), PNI (OR = 0.96, 95% CI: 0.93–0.98, *P* = 0.001) and GNRI (OR = 0.96, 95% CI: 0.94–0.98, *P* < 0.001) were significantly negatively associated with DR risk, whereas ALBI (OR = 1.73, 95% CI: 1.17–2.56, *P* = 0.006) and NPAR (OR = 1.05, 95% CI: 1.01–1.09, *P* = 0.013) showed significant positive associations. When categorized by tertiles, compared with the lowest tertile, the highest tertile of PNI was associated with a 43% lower DR risk (OR = 0.57, 95% CI: 0.41–0.80, *P* = 0.001), and the highest GNRI tertile with a 44% lower risk (OR = 0.56, 95% CI: 0.41–0.76, *P* < 0.001). Conversely, the highest ALBI tertile showed a 58% higher risk (OR = 1.58, 95% CI: 1.18–2.19, *P* = 0.003), and the highest NPAR tertile a 37% higher risk (OR = 1.37, 95% CI: 1.02–1.83, *P* = 0.036). These associations remained consistent across Models 1 to 3, and all *P* for trend across tertiles were < 0.05, indicating significant dose-response relationships.

**Table 3:** Association of nutritional-inflammatory-related indices with the prevalence of DR in NHANES cohort.

Variable	Model 1		Model 2		Model 3	
	OR (95% CI)	*P* value	OR (95% CI)	*P* value	OR (95% CI)	*P* value
PNI						
Continuous	0.95 (0.93, 0.97)	**<0.001**	0.95 (0.93, 0.97)	**<0.001**	0.96 (0.93, 0.98)	**0.001**
Q1	1.00 (Reference)		1.00 (Reference)		1.00 (Reference)	
Q2	0.53 (0.40, 0.70)	**<0.001**	0.53 (0.40, 0.71)	**<0.001**	0.56 (0.41, 0.75)	**<0.001**
Q3	0.54 (0.41, 0.72)	**<0.001**	0.53 (0.39, 0.72)	**<0.001**	0.57 (0.41, 0.80)	**0.001**
* P* for trend		**<0.001**		**<0.001**		**<0.001**
ALBI						
Continuous	2.27(1.56, 3.29)	**<0.001**	2.25 (1.56, 3.24)	**<0.001**	1.73 (1.17, 2.56)	**0.006**
Q1	1.00 (Reference)		1.00 (Reference)		1.00 (Reference)	
Q2	1.51 (1.12, 2.02)	**0.007**	1.55 (1.15, 2.08)	**0.004**	1.53 (1.14, 2.06)	**0.006**
Q3	1.79 (1.32, 2.41)	**<0.001**	1.81 (1.35, 2.44)	**<0.001**	1.58 (1.18, 2.19)	**0.003**
* P* for trend		**<0.001**		**<0.001**		**<0.01**
NPAR						
Continuous	1.08 (1.04, 1.11)	**<0.001**	1.08 (1.04, 1.12)	**<0.001**	1.05 (1.01, 1.09)	**0.013**
Q1	1.00 (Reference)		1.00 (Reference)		1.00 (Reference)	
Q2	1.10 (0.85, 1.42)	0.471	1.13 (0.86, 1.49)	0.362	1.10 (0.83, 1.46)	0.515
Q3	1.50 (1.18, 1.91)	**0.001**	1.56 (1.21, 2.01)	**<0.001**	1.37 (1.02, 1.83)	**0.036**
* P* for trend		**<0.01**		**<0.001**		**<0.05**
GNRI						
Continuous	0.95 (0.93, 0.97)	**<0.001**	0.95(0.93, 0.97)	**<0.001**	0.96 (0.94, 0.98)	**<0.001**
Q1	1.00 (Reference)		1.00 (Reference)		1.00 (Reference)	
Q2	0.65 (0.48, 0.87)	**0.004**	0.64 (0.48, 0.86)	**0.004**	0.69 (0.51, 0.93)	**0.015**
Q3	0.51 (0.38, 0.69)	**<0.001**	0.50 (0.37, 0.66)	**<0.001**	0.56 (0.41, 0.76)	**<0.001**
* P* for trend		**<0.001**		**<0.001**		**<0.001**

Model 1 was adjusted for none. Model 2 was adjusted for age, sex, race, marital status, education level. Model 3 was adjusted for Model 2 variables plus smoking status, drinking status, BMI, HbA1c, eGFR, hypertension, hyperlipidemia.

CI, confidence interval; DR, diabetic retinopathy; OR, odds ratio; PNI, prognostic nutritional index; NPAR, neutrophil percentage to albumin ratio; ALBI, albumin-bilirubin; GNRI, geriatric nutritional risk index.

The bold values indicate statistically significant results (P < 0.05).

The hospital-based clinical cohort further strengthened these findings, with more pronounced effect sizes. As presented in **Table 4** and **Figure 2**. In the fully adjusted model, PNI (OR = 0.85, 95% CI: 0.79–0.91, *P* < 0.001) and GNRI (OR = 0.80, 95% CI: 0.74–0.86, *P* < 0.001) remained significantly negatively associated with DR risk, whereas ALBI (OR = 32.80, 95% CI: 9.35–137.00, *P* < 0.001) and NPAR (OR = 1.17, 95% CI: 1.07–1.30, *P* = 0.003) showed positive associations. Tertile analysis revealed that compared with the lowest tertile, the highest PNI tertile was associated with an 85% lower DR risk (OR = 0.15, 95% CI: 0.06–0.32, *P* < 0.001), and the highest GNRI tertile with a 95% lower risk (OR = 0.05, 95% CI: 0.02–0.12, *P* < 0.001). Conversely, the highest ALBI tertile had a 9.66-fold higher risk (OR = 9.66, 95% CI: 4.30–23.2, *P* < 0.001), and the highest NPAR tertile a 4.36-fold higher risk (OR = 4.36, 95% CI: 2.02–9.79, *P* < 0.001). All *P* for trend across tertiles were < 0.001, clearly indicating a dose-response relationship between nutritional-inflammatory status and DR risk. These results indicate that lower nutritional reserves and higher inflammatory burdens jointly point to an increased risk of DR.

**Table 4:** Association of nutritional-inflammatory-related indices with the prevalence of DR in hospital-based clinical cohort.

Variable	Model 1		Model 2		Model 3	
	OR (95% CI)	*P* value	OR (95% CI)	*P* value	OR (95% CI)	*P* value
PNI						
Continuous	0.85 (0.80, 0.90)	**<0.001**	0.84 (0.79, 0.90)	**<0.001**	0.85 (0.79, 0.91)	**<0.001**
Q1	1.00 (Reference)		1.00 (Reference)		1.00 (Reference)	
Q2	0.25 (0.13, 0.47)	**<0.001**	0.24 (0.12, 0.46)	**<0.001**	0.26 (0.13, 0.53)	**<0.001**
Q3	0.14 (0.07, 0.28)	**<0.001**	0.14 (0.07, 0.29)	**<0.001**	0.15 (0.06, 0.32)	**<0.001**
* P* for trend		**<0.001**		**<0.001**		**<0.001**
ALBI						
Continuous	45.00 (13.70,172.00)	**<0.001**	46.30 (13.60, 186.00)	**<0.001**	32.8 (9.35, 137.00)	**<0.001**
Q1	1.00 (Reference)		1.00 (Reference)		1.00 (Reference)	
Q2	2.55 (1.20, 5.67)	**0.017**	2.38 (1.10, 5.41)	**0.032**	2.60 (1.13, 6.24)	**0.027**
Q3	11.00 (5.34, 24.4)	**<0.001**	10.9 (5.09, 24.9)	**<0.001**	9.66 (4.30, 23.2)	**<0.001**
* P* for trend		**<0.001**		**<0.001**		**<0.001**
NPAR						
Continuous	1.24 (1.13, 1.38)	**<0.001**	1.23 (1.12, 1.36)	**<0.001**	1.17 (1.07, 1.30)	**0.003**
Q1	1.00 (Reference)		1.00 (Reference)		1.00 (Reference)	
Q2	1.58 (0.78, 3.26)	0.210	1.45 (0.70, 3.08)	0.322	1.34 (0.61, 2.98)	0.471
Q3	5.94 (3.04, 12.1)	**<0.001**	5.51 (2.71, 11.7)	**<0.001**	4.36 (2.02, 9.79)	**<0.001**
* P* for trend		**<0.001**		**<0.001**		**<0.001**
GNRI						
Continuous	0.81 (0.75, 0.86)	**<0.001**	0.79 (0.73, 0.85)	**<0.001**	0.80 (0.74, 0.86)	**<0.001**
Q1	1.00 (Reference)		1.00 (Reference)		1.00 (Reference)	
Q2	0.22 (0.11, 0.42)	**<0.001**	0.17 (0.08, 0.33)	**<0.001**	0.18 (0.08, 0.39)	**<0.001**
Q3	0.05 (0.02, 0.12)	**<0.001**	0.04 (0.02, 0.10)	**<0.001**	0.05 (0.02, 0.12)	**<0.001**
* P* for trend		**<0.001**		**<0.001**		**<0.001**

Model 1 was adjusted for none. Model 2 was adjusted for age, sex, marital status, education level. Model 3 was adjusted for Model 2 variables plus smoking status, drinking status, BMI, HbA1c, eGFR, hypertension, hyperlipidemia.

*CI*, confidence interval; *OR*, odds ratio; *PNI*, prognostic nutritional index; *NPAR*, neutrophil percentage to albumin ratio; *ALBI*, albumin-bilirubin; *GNRI*, geriatric nutritional risk index. The bold values indicate statistically significant results (P < 0.05).

### 3.3 Restricted cubic splines of the association between nutritional-inflammatory-related indices and DR

**Figure 3:**
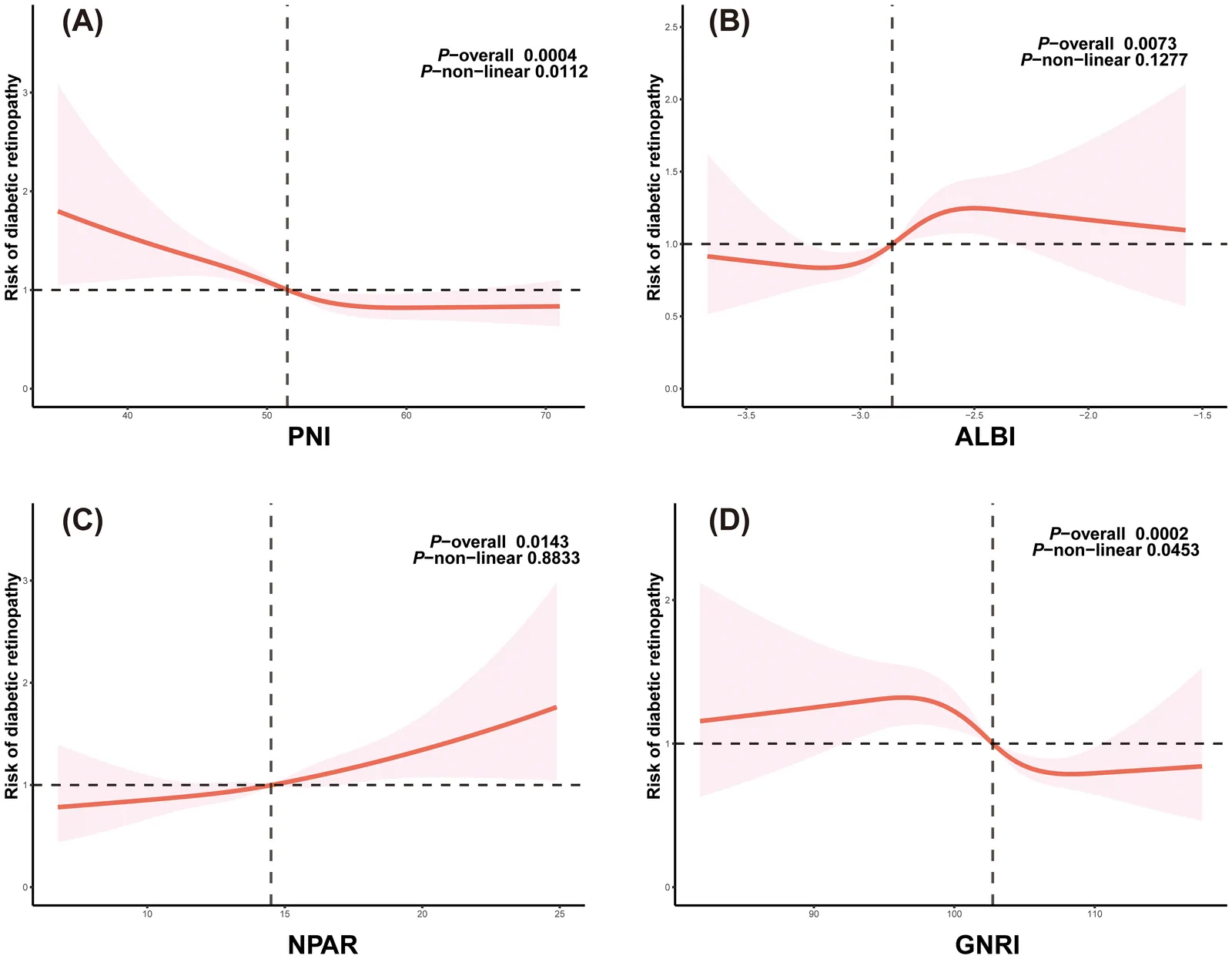
**(A)** RCS analysis of the association between PNI and DR; **(B)** RCS analysis of the association between ALBI and DR; **(C)** RCS analysis of the association between NPAR and DR; **(D)** RCS analysis of the association between GNRI and DR. DR, diabetic retinopathy.

**Figure 4:**
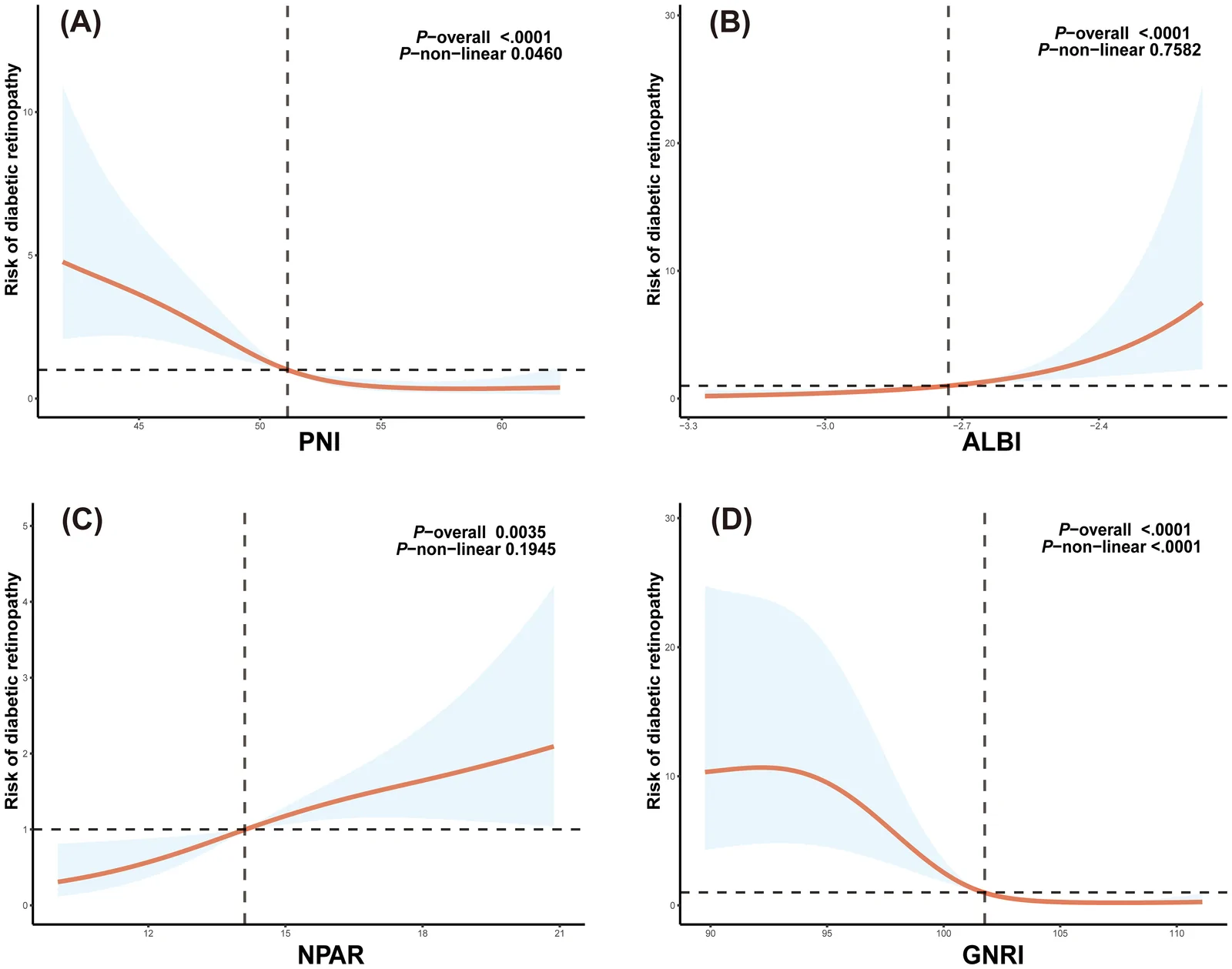
**(A)** RCS analysis of the association between PNI and DR; **(B)** RCS analysis of the association between ALBI and DR; **(C)** RCS analysis of the association between NPAR and DR; **(D)** RCS analysis of the association between GNRI and DR. DR, diabetic retinopathy.

To explore potential nonlinear associations between nutritional-inflammatory indices and DR, we performed RCS analysis. In the NHANES cohort, after multivariable adjustment, ALBI and NPAR showed linear relationships with DR (*P* for nonlinearity > 0.05), whereas PNI and GNRI demonstrated nonlinear relationships (*P* for nonlinearity < 0.05) (**Figure 3**). Consistently, similar results were observed in the hospital-based clinical cohort (**Figure 4**).

### 3.4 Threshold effects of PNI and GNRI on DR

The RCS curves revealed nonlinear relationships between PNI, GNRI, and DR. To identify key inflection points, we further performed threshold effect analysis. As shown in **Table 5**. In the NHANES cohort, the inflection points for PNI and GNRI were 56.00 and 98.27, respectively. When PNI < 56, PNI showed a significant negative association with DR (OR = 0.960, *P* < 0.001); beyond this threshold, the association was no longer significant. For GNRI, a significant protective effect was observed only when GNRI > 98.27 (OR = 0.957, *P* < 0.001). However, the overall likelihood ratio test (*P* = 0.101) suggested weak evidence for a threshold effect of GNRI in the U.S. population.

**Table 5:** Threshold effects of PNI and GNRI on DR.

Data source (indicator)	Outcome	Effect	*P* value
NHANES cohort (PNI)			
	Inflection point	56.00	
	<56.00	0.960 (0.941-0.978)	<0.001
	>56.00	1.001 (0.978-1.017)	0.903
	P for likelihood test		0.014
NHANES cohort (GNRI)			
	Inflection point	98.27	
	<98.27	0.999 (0.961-1.039)	0.971
	>98.27	0.957 (0.935-0.980)	<0.001
	P for likelihood test		0.101
Clinical cohort (PNI)			
	Inflection point	42.85	
	<42.85	1.052 (0.883-1.285)	0.543
	>42.85	0.828 (0.768-0.886)	<0.001
	P for likelihood test		0.022
Clinical cohort (GNRI)			
	Inflection point	90.39	
	<90.39	1.136 (0.981-1.61)	0.195
	>90.39	0.761 (0.695-0.825)	<0.001
	P for likelihood test		<0.001

This table presents the inflection point analysis of PNI and GNRI based on NHANES cohort and clinical cohort. The inflection point indicates the value at which the association with DR changes significantly. For each indicator and cohort, the effect size (OR) and 95% CI are shown for values below and above the inflection point, as well as the p-values for the likelihood ratio test to assess model fit.

DR, diabetic retinopathy; PNI, prognostic nutritional index; GNRI, geriatric nutritional risk index.

In the hospital-based clinical cohort, the inflection points for PNI and GNRI were 42.85 and 90.39, respectively. PNI > 42.85 was significantly negatively associated with DR (OR = 0.828, P < 0.001), and GNRI > 90.39 also reduced DR risk (OR = 0.957, P < 0.001). The overall likelihood ratio test (P < 0.001) strongly supported a threshold effect for GNRI. These findings indicate that the impact of PNI and GNRI on DR differs significantly across their levels and that the inflection point values vary by data source.

### 3.5 Stratified analysis and subgroup analysis

**Figure 5:**
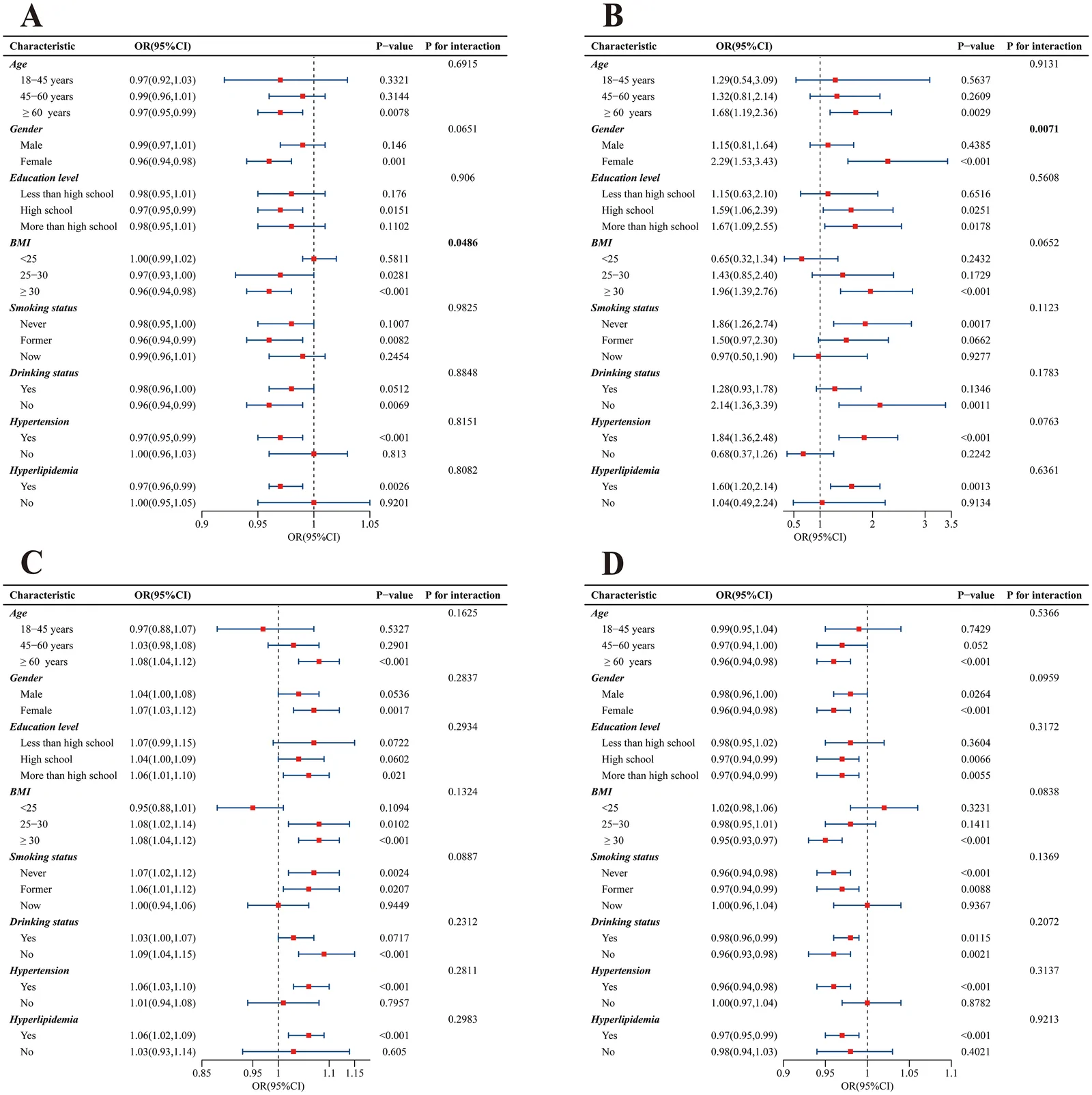
Subgroup analyses for the association between nutritional-inflammatory-related indices and DR in the NHANES cohort. **(A)** Subgroup analysis of the association between PNI and DR; **(B)** Subgroup analysis of the association between ALBI and DR; **(C)** Subgroup analysis of the association between NPAR and DR; **(D)** Subgroup analysis of the association between GNRI and DR. BMI, body mass index; DR, diabetic retinopathy; PNI, prognostic nutritional index; NPAR, neutrophil percentage to albumin ratio; ALBI, albumin-bilirubin; GNRI, geriatric nutritional risk index. The bold values indicate statistically significant results (P < 0.05).

To examine effect modification by nutritional-inflammatory-related indices, we performed stratified analyses in the NHANES cohort based on age, sex, education level, BMI, smoking, alcohol use, hypertension, and hyperlipidemia. As shown in **Figure 5**, the associations of NPAR and GNRI with DR were not modified by these stratification factors (all P for interaction > 0.05). However, we observed two significant interactions: between PNI and BMI groups (*P* for interaction = 0.049) and between ALBI and sex (*P* for interaction = 0.007). Among subgroups with significant interactions, the protective effect of PNI was more pronounced in overweight/obese individuals (BMI > 25 kg/m²), suggesting that improving nutritional and inflammatory status may provide greater clinical benefit for DR prevention in this population. The positive correlation between ALBI and DR was markedly more pronounced in female patients, suggesting that diabetic women necessitate more vigilant monitoring of this liver function and inflammatory marker.

### 3.6 Analysis of the relationship between nutritional-inflammatory-related indices and DR severity in the hospital-based clinical cohort

**Figure 6:**
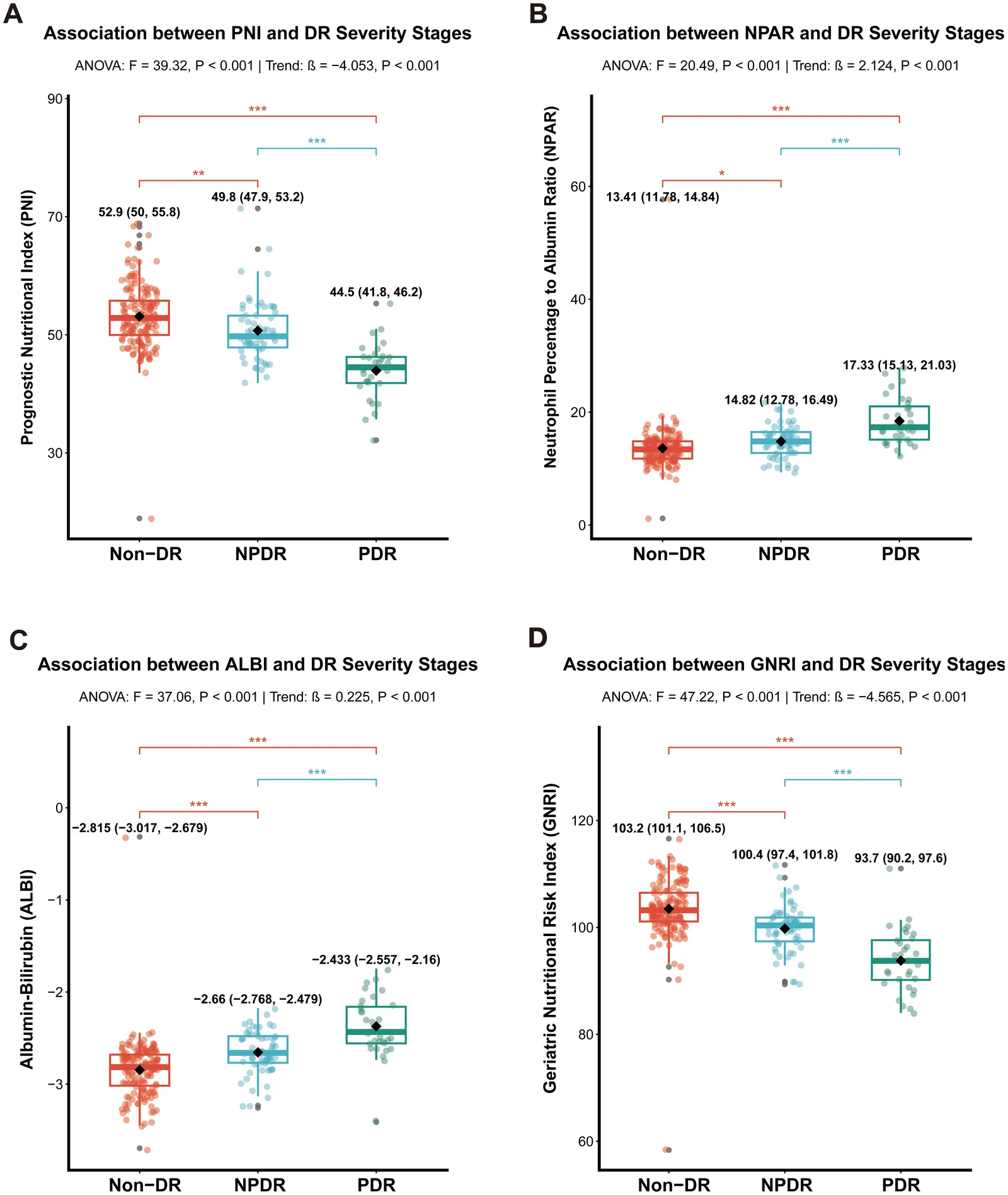
Levels of nutritional-inflammation-related indices across different DR severity stages in the hospital-based clinical cohort. ANOVA, analysis of variance; DR, diabetic retinopathy; Non-DR, Non-diabetic retinopathy; NPDR, nonproliferative diabetic retinopathy; PDR, proliferative diabetic retinopathy; PNI, prognostic nutritional index; NPAR, neutrophil percentage to albumin ratio; ALBI, albumin-bilirubin; GNRI, geriatric nutritional risk index. **(A)** PNI levels across different DR severity stages; **(B)** NPAR levels across different DR severity stages; **(C)** ALBI levels across different DR severity stages; **(D)** GNRI levels across different DR severity stages. *P < 0.05, **P < 0.01, ***P < 0.001 vs. the corresponding control group.

In the hospital-based clinical cohort, to elucidate the dose-response relationship between nutritional-inflammatory indices and DR severity, all four indices showed significant trends across the no DR, NPDR, and PDR groups. Mean PNI and GNRI decreased with increasing DR severity (both *P* for trend < 0.001), reinforcing their inverse association with DR progression. Conversely, mean ALBI and NPAR increased with DR severity (both *P* for trend < 0.001), confirming their positive association with DR progression. As presented in **Figure 6**.

### 3.7 Sensitivity analysis

To assess the robustness of our findings, a series of sensitivity analyses were conducted in the NHANES cohort (**Supplementary Tables 2–4**). First, to evaluate the impact of sampling weights, unweighted logistic regression models were applied. The results were consistent with the primary analysis, showing that ALBI and NPAR remained positively associated with DR risk, whereas PNI and GNRI maintained significant negative associations. Second, to reduce bias due to extreme values in nutritional and inflammatory indices, we repeated the weighted logistic regression analysis after excluding outliers. The findings were essentially unchanged, further supporting the robustness of our conclusions. Finally, to further account for potential confounding from liver function, hematological indices, and socioeconomic status, we additionally adjusted for alanine transaminase (ALT), aspartate transaminase (AST), red blood cell distribution width (RDW), and the family income-to-poverty ratio (PIR) in the final model. The results remained consistent with the primary analysis, further supporting the robustness of our findings.

## 4 Discussion

The pathogenesis of DR involves complex interactions among multiple pathological processes. While hyperglycemia is widely recognized as a key driver of DR risk and progression, HbA1c and disease duration account for only about 11% of the risk of microvascular complications, and DR may still develop despite good glycemic control (4, 24–26). In recent years, the role of nutritional status and inflammatory burden in DR management has attracted growing attention, as decreased serum albumin levels and various inflammatory cytokines are strongly linked with increased DR risk and disease progression (27–29). Given the limitations of single biomarkers, composite indicators that integrate nutritional and inflammatory information hold significant research value for elucidating the pathogenesis of DR and uncovering non-glucose-dependent pathogenic pathways. Such composite indicators have demonstrated promising predictive value in chronic systemic diseases, including diabetic foot ulcer, type 1 diabetes prognosis, nephropathy, depression, and malignancies (16, 30–32); however, studies focusing on DR remain fragmented, and robust replication analyses using independent hospital-based clinical cohorts are generally lacking.

This dual-cohort study, leveraging the nationwide NHANES population and an independent hospital-based clinical cohort, is the first to systematically investigate the associations between four nutritional-inflammation-related indices (PNI, GNRI, ALBI, and NPAR) and DR. Our analysis revealed that PNI and GNRI were independently and negatively associated with DR risk, whereas ALBI and NPAR were independently and positively associated with DR risk, all exhibiting clear dose–response relationships. RCS analyses revealed linear associations of ALBI and NPAR with DR, but non-linear relationships between PNI, GNRI and DR; threshold effect analyses further identified their critical inflection points. Subgroup analyses indicated that the protective association of PNI was more prominent in overweight/obese individuals, whereas the association of ALBI with DR risk was stronger in females. Importantly, findings from the supplementary clinical cohort were highly consistent with the above results and showed more pronounced effects. They further confirmed that these indicators were progressively associated with DR severity, thereby strengthening the robustness and generalizability of the conclusions in real-world settings. In summary, results from this dual-cohort validation suggest that PNI, GNRI, ALBI, and NPAR play important roles in the development and progression of DR. As easily accessible routine laboratory parameters, these indicators show potential as effective tools for risk stratification and early detection of DR.

PNI was negatively associated with DR. Originally proposed by Buzby et al. (33) to predict outcomes in gastrointestinal surgery, PNI integrates serum albumin and peripheral lymphocyte count, thus reflecting nutritional and immune-inflammatory status. Our findings align with recent studies (34, 35) and, for the first time across two independent cohorts, reveal a nonlinear relationship and threshold effect between PNI and DR. External validation further confirmed its association with DR progression, suggesting that PNI may serve as both risk prediction and disease stratification. Notably, the inflection points differed between the two cohorts (NHANES: 56.00; validation cohort: 42.85), with opposite association patterns on either side of the thresholds. Beyond differences in ethnicity and sample size, systematic variations in nutritional-inflammatory burden between the cohorts may better explain this discrepancy. The NHANES cohort is derived from a community-dwelling population with generally good nutritional reserves; among diabetic patients in this cohort, maintaining a PNI above 56.00 did not confer additional benefit. In contrast, the clinical cohort consisted of hospitalized patients who experienced greater disease−related nutritional depletion and inflammatory burden, and the protective association was more pronounced within the lower PNI range. For these hospitalized patients with evident nutritional depletion, raising the PNI from extremely low levels to above 42.85 was associated with a significant reduction in DR risk. These two thresholds are not contradictory but rather apply to different disease severity strata—community versus inpatient settings. Together, these findings suggest that nutritional intervention targets should be tailored according to the patient’s baseline nutritional status and disease burden, rather than applying a single uniform standard across all populations.

Several mechanisms may explain the protective association between PNI and DR. First, higher serum albumin was inversely associated with DR risk and progression. Previous experimental studies have demonstrated that albumin exerts inhibitory effects on reactive oxygen species (ROS), nuclear factor-κB (NF-κB), and NLRP3 inflammasome activation, with consequent reductions in pro-inflammatory cytokines such as tumor necrosis factor-α (TNF-α), interleukin-6 (IL-6), and interleukin-1β (IL-1β)—all of which are closely implicated in DR pathogenesis (36, 37). In addition, oxidative stress is common in patients with type 2 diabetes mellitus. Albumin has been associated with antioxidant properties, including scavenging reactive oxygen and nitrogen species and binding glycation products, which may correlate with reduced retinal oxidative damage (38, 39). Second, hypoalbuminemia was inversely associated with optic disc edema and accelerated retinal arteriovenous flow, worsening microvascular damage and leakage—hallmarks of DR—whereas adequate albumin maintains vascular integrity and barrier function (40, 41). Furthermore, albumin binds advanced glycation end-products (AGEs), reducing their direct retinal toxicity (42, 43). The lymphocyte component of PNI reflects inflammatory immune status. Studies have shown that the ratio of CD4+CD25hiTreg to Th17 or Th1 cells is reduced in patients with type 2 diabetes, promoting inflammatory activation and complications (44). Elevated neutrophil-to-lymphocyte ratio (NLR) is also associated with increased DR risk, indicating that inflammatory immune activation plays a key role in DR pathogenesis (45). Therefore, PNI—an integrative index combining albumin and lymphocyte count—reflects both nutritional and inflammatory status, and its inverse association with DR may be explained by its potential anti-inflammatory and vascular protective properties. Nevertheless, the specific pathways linking PNI to DR remain unclear and warrant further investigation.

GNRI exhibited a consistent protective association with DR across both cohorts. GNRI, which integrates serum albumin and BMI to assess geriatric nutritional risk, sensitively reflects nutritional depletion in chronic disease patients (46). Horinouchi et al. (47) linked lower GNRI to heightened complication risk in 204 patients with type 2 diabetes. Similarly, Cho et al. (48) reported an inverse GNRI–DR association in a Korean cohort and found that GNRI surpassed BMI in predicting DR. The aforementioned studies were limited to Asian populations; however, the present study not only validated the negative correlation between GNRI and DR across ethnic groups but also, for the first time, uncovered a nonlinear relationship through RCS and threshold analysis. Clinical cohort further corroborated the association of GNRI with DR progression, thereby broadening its utility in risk stratification. Notably, a significant threshold effect emerged solely in the clinical cohort, with no such effect detected in NHANES (*P* = 0.101). This discrepancy may be attributed to population differences: NHANES is community-based with generally better nutritional status, whereas the clinical cohort consisted of clinical patients with greater fluctuations in albumin and BMI and a higher proportion of extreme low values, making the threshold effect more detectable. In the clinical cohort, the GNRI cut-off of 90.39 demarcated a threshold above which better nutritional status was associated with lower DR prevalence, offering a potential target for clinical nutritional intervention.

Potential mechanisms linking low GNRI to elevated DR risk are outlined below. Comprising serum albumin and body weight, GNRI is less confounded by extracellular fluid shifts than either component alone. Therefore, interpreting its protective association against DR using only a single indicator lacks systematicity and comprehensiveness (49). Although both PNI and GNRI reflect nutritional status, they capture different dimensions: PNI represents nutrition-inflammation immune balance, whereas GNRI focuses on protein-energy wasting, with low GNRI typically indicating systemic malnutrition (50, 51). Malnutrition exacerbates microinflammation, elevating pro-inflammatory cytokines such as TNF-α and IL-6, which are closely linked to DR progression (52–54). Furthermore, malnourished patients exhibit increased nicotinamide adenine dinucleotide phosphate(NADPH) oxidase activity, promoting superoxide anion production in endothelial cells, which inactivates nitric oxide and leads to endothelium-dependent diastolic dysfunction and endothelial injury (55, 56), and endothelial dysfunction represents a key pathological feature of DR (57). Thus, low GNRI is associated with DR risk, potentially via microinflammatory and endothelial pathways. Nonetheless, the precise molecular pathways connecting GNRI to DR warrant further elucidation.

The ALBI score was positively associated with DR risk. The ALBI score integrates albumin and bilirubin to reflect liver functional reserve. Originally developed for assessing liver function in hepatocellular carcinoma as an alternative to the Child–Pugh grade, the ALBI score has now been widely used to evaluate systemic inflammation and metabolic disorders (29, 58–60). This study is the first to demonstrate a robust linear association between ALBI score and DR in a large, nationally representative cohort with clinical replication. This finding aligns with prior evidence of an inverse association between albumin and DR. Hypoalbuminemia reduces antioxidant capacity and impairs endothelial barrier integrity, both of which are central to the pathogenesis of DR. Furthermore, the ALBI score incorporates bilirubin, a controversial marker, which may provide a more comprehensive risk assessment than albumin alone. Some studies have suggested that bilirubin, as an endogenous antioxidant, reduces DR risk (61–63). Recent evidence suggests a U-shaped relationship, wherein physiological concentrations confer protection, yet excessive levels may elevate DR risk through oxidative stress and inflammatory pathways (64, 65).

An elevated ALBI score is associated with liver dysfunction, and diminished clearance of metabolic waste products like ammonia may contribute to endothelial dysfunction, a fundamental pathological mechanism of DR (57, 66). In addition, the ALBI score serves as an integrated marker of systemic inflammatory burden, as both hyperbilirubinemia and hypoalbuminemia exacerbate inflammation, which in turn suppresses albumin synthesis and promotes protein catabolism, creating a vicious cycle (67, 68). Persistent low-grade inflammation is a core driver of DR, and this cycle may explain the observed association between elevated ALBI and DR progression in the clinical cohort. Of note, elevated bilirubin may exert amplified toxic effects in the setting of hypoalbuminemia (69). Consequently, secondary hyperbilirubinemia due to impaired liver function is often accompanied by reduced albumin and activated inflammation, under which systemic inflammation and endothelial injury may override the antioxidant protection of physiological bilirubin, collectively associated with an increased risk of DR. In summary, by integrating two critical hepatic parameters—albumin and bilirubin—the ALBI score provides a robust instrument for DR risk stratification, though the precise mechanistic underpinnings merit further exploration.

This study identified a significant positive association between NPAR and DR risk. NPAR, calculated as the ratio of neutrophil percentage to albumin, reflects a dual unfavorable status of enhanced inflammation and reduced antioxidant capacity (70). Previous studies have shown good predictive value of NPAR in metabolic syndrome, chronic kidney disease, and periodontitis (71–73). Using two cohorts, this study is the first to systematically confirm a positive association between NPAR and DR risk in a large multi-ethnic population, consistent with recent literature (73). Additionally, NPAR has been implicated in diabetic kidney disease, suggesting its potential as a broad-spectrum marker for diabetic microvascular complications (74). The clinical cohort in this study further supports its clinical utility in DR risk stratification and disease assessment.

The precise mechanisms linking elevated NPAR to increased DR risk remain incompletely defined, though current evidence implicates several pathways. First, elevated NPAR derives from increased neutrophil percentage and/or hypoalbuminemia, the latter being consistently associated with greater DR risk (36, 37, 75). Second, diabetic microvascular complications frequently coincide with neutrophilia, indicative of chronic low-grade inflammation that exacerbates retinal microvascular injury (76). Critically, under high-glucose conditions, neutrophil extracellular trap (NET) formation is increased and has been linked to endothelial damage, which may contribute to retinal microvascular injury (77, 78). Moreover, serum albumin suppresses neutrophil activation marker expression and reactive oxygen species generation (79). Thus, as the ratio of neutrophil percentage to albumin, NPAR more comprehensively reflects the dual status of nutritional status and inflammatory burden, offering unique integrated value in DR risk assessment (80).

Subgroup analyses revealed two significant interaction effects. First, PNI interacted with BMI, with its protective association against DR being more pronounced in overweight/obese individuals (BMI ≥25 kg/m²). This finding differs from the independent association reported by Yang et al. (81), likely attributable to their use of a BMI cutoff ≥24 kg/m² in an Asian cohort, which may have diminished stratification discrimination and attenuated the interaction effect. Biologically, overweight/obese individuals exhibit a heightened systemic inflammatory burden stemming from adipose tissue-derived pro-inflammatory cytokines (TNF-α, IL-6, IL-1β), thereby amplifying the benefits conferred by PNI enhancement (82). This suggests that actively improving PNI may offer superior DR prevention value in overweight/obese diabetic patients. Second, the positive association between ALBI and DR was significantly stronger in female patients, indicating heightened sensitivity to hepatic function-related nutritional-inflammatory dysregulation among diabetic women. This sex difference may be attributed to estrogen regulation of bilirubin metabolism, higher baseline inflammatory levels in women with type 2 diabetes, and relatively lower albumin synthesis capacity (83–85). Therefore, DR screening strategies should incorporate sex-specific ALBI thresholds, and even mildly elevated ALBI in female patients warrants vigilance for DR onset and progression. However, it should be noted that the subgroup analysis examined multiple stratifying variables, which may entail a certain risk of false positives; nevertheless, this still provides directions for personalized DR risk stratification and intervention.

This study has several notable strengths. First, the dual-cohort design combining the nationally representative NHANES database with an independent hospital-based clinical cohort ensures both national generalizability and mitigates the limitations of self-reported DR diagnosis, thereby enhancing the robustness and applicability of the findings. Second, the clinical replication cohort employed objective examinations to determine DR severity, confirming that nutritional-inflammatory-related indicators can distinguish between mild and severe DR, which underscores their clinical utility. Furthermore, the integrated use of RCS, threshold effect analysis, subgroup interaction testing, and multiple sensitivity analyses thoroughly elucidates the complex associations and reliability of these indicators with DR. Finally, the PNI and GNRI thresholds identified in this study show potential for clinical translation. These indices can be automatically calculated from routine blood counts and biochemistry panels at no additional cost, facilitating seamless integration into laboratory reporting systems for diabetes care.

This study has several limitations that warrant consideration. First, although an independent clinical replication cohort was included, both datasets derive from cross-sectional designs, thereby precluding causal inference. Second, the DR assessment in NHANES relies on self-reporting, which inevitably introduces recall bias and bidirectional misclassification bias, although we attempted to compensate for this limitation by using objective examinations in the clinical cohort. Third, to maintain sufficient statistical power, we did not completely adjust for residual confounding; therefore, the findings may be influenced by unmeasured confounders, including diabetes duration. Fourth, NHANES reflects the non-institutionalized U.S. population and the Chinese clinical cohort was limited in size, necessitating caution when extrapolating these findings. Fifth, our clinical cohort—being single-center and hospital-based with a limited sample size—precludes external validation; larger multicenter cohorts are warranted to corroborate our results. Finally, all indices were assessed at a single time point and thus cannot capture long-term dynamic influences on DR. Longitudinal studies with repeated measurements are needed to delineate temporal associations between these indicators and DR.

## 5 Conclusion

In summary, utilizing a dual-validation framework comprising the U.S. NHANES database and a Chinese hospital-based clinical cohort, this study demonstrates that PNI and GNRI exhibit independent inverse associations with DR risk, whereas ALBI and NPAR show independent positive associations. Furthermore, the hospital-based clinical cohort reveals significant correlations between these indices and DR severity. These findings highlight the critical value of incorporating nutritional and inflammatory status into DR risk assessment. Given that PNI, ALBI, GNRI, and NPAR are readily derivable from routine laboratory parameters, they represent pragmatic and promising tools for DR risk stratification and early warning, although further validation in prospective studies is warranted.

## Data Availability

The datasets presented in this study can be found in online repositories. The names of the repository/repositories and accession number(s) can be found below: https://www.cdc.gov/nchs/nhanes/.
