# Supplementary material for: Association between nutritional‑inflammatory status and diabetic retinopathy in adults with diabetes: insights from a US population‑based survey and a Chinese hospital‑based clinical cohort
Source: Front Endocrinol (Lausanne). 2026 Aug 6;17:1883581. doi: 10.3389/fendo.2026.1883581 (PMC13489890; doi:10.3389/fendo.2026.1883581)
Supplement: Supplementary file 1 [file Supplementaryfile1.docx]

**SUPPLEMENTAL MATERIAL**

***Supplementary Tables***

**Table S1** STROBE Checklist.

**Table S2** Unweighted logistic regression analysis of the association between nutritional-inflammatory-related indices and DR prevalence in the NHANES.

**Table S3** The association between nutritional-inflammatory-related indices and DR prevalence after outlier exclusion in the NHANES.

**Table S4** Association of nutritional-inflammatory-related indices with DR prevalence after additional adjustment for potential confounders in the NHANES.

***Supplementary Figure***

**Figure S1.** Spearman correlation heatmap among the four nutrition‑inflammation‑related indices in the NHANES cohort.

**Table S1 STROBE Checklist**

|  | Item No | Recommendation | Page /Paragraph |
| --- | --- | --- | --- |
| **Title and abstract** | 1 | (*a*) Indicate the study’s design with a commonly used term in the title or the abstract | Page 1. Title: Association between nutritional- inflammatory status and diabetic retinopathy in adults with diabetes: findings from a US population-based survey and a Chinese hospital-based clinical cohort |
|  |  | (*b*) Provide in the abstract an informative and balanced summary of what was done and what was found | Page 1-2. Abstract includes background, methods, results, and conclusions. |
| Introduction | | |  |
| Background/rationale | 2 | Explain the scientific background and rationale for the investigation being reported | Page 2. Introduction includes the burden of DR, the association between nutritional-inflammatory-related indices and DR, and the advantages of nutritional-inflammatory-related indices. |
| Objectives | 3 | State specific objectives, including any prespecified hypotheses | Page 2. Introduction: The final paragraph |
| Methods | | |  |
| Study design | 4 | Present key elements of study design early in the paper | Page 2-3. Methods: 2.1 NHANES cohort and 2.2 Hospital-based clinical cohort |
| Setting | 5 | Describe the setting, locations, and relevant dates, including periods of recruitment, exposure, follow-up, and data collection | Page 2-3. Methods: 2.1 NHANES cohort and 2.2 Hospital-based clinical cohort |
| Participants | 6 | (*a*) Give the eligibility criteria, and the sources and methods of selection of participants | Page 2-3. Methods: 2.1 NHANES cohort and 2.2 Hospital-based clinical cohort |
| Variables | 7 | Clearly define all outcomes, exposures, predictors, potential confounders, and effect modifiers. Give diagnostic criteria, if applicable | Page 3-4. Methods: 2.3 Exposure variables and outcome definition; 2.4 Covariates |
| Data sources/ measurement | 8* | For each variable of interest, give sources of data and details of methods of assessment (measurement). Describe comparability of assessment methods if there is more than one group | Page 2-4. Methods: 2.1 NHANES cohort; 2.2 Hospital-based clinical cohort; 2.3 Exposure variables and outcome definition; 2.4 Covariates |
| Bias | 9 | Describe any efforts to address potential sources of bias | Page 4. Methods: 2.6 Statistical analysis |
| Study size | 10 | Explain how the study size was arrived at | Page 2-3. Methods: 2.1 NHANES cohort and 2.2 Hospital-based clinical cohort |
| Quantitative variables | 11 | Explain how quantitative variables were handled in the analyses. If applicable, describe which groupings were chosen and why | Page 4. Methods: 2.6 Statistical analysis |
| Statistical methods | 12 | (*a*) Describe all statistical methods, including those used to control for confounding | Page 4. Methods: 2.6 Statistical analysis |
|  |  | (*b*) Describe any methods used to examine subgroups and interactions | Page 4. Methods: 2.6 Statistical analysis |
|  |  | (*c*) Explain how missing data were addressed | Page 2-3. Methods: 2.1 NHANES cohort and 2.2 Hospital-based clinical cohort |
|  |  | (*d*) If applicable, describe analytical methods taking account of sampling strategy | Page 4. Methods: 2.6 Statistical analysis |
|  |  | (*e*) Describe any sensitivity analyses | Page 4. Methods: 2.6 Statistical analysis |
| Results | | |  |
| Participants | 13* | (a) Report numbers of individuals at each stage of study—eg numbers potentially eligible, examined for eligibility, confirmed eligible, included in the study, completing follow-up, and analysed | Page 5. Results: 3.1 Baseline characteristics |
|  |  | (b) Give reasons for non-participation at each stage | Refer to "FIGURE 1" |
|  |  | (c) Consider use of a flow diagram | Refer to "FIGURE 1" |
| Descriptive data | 14* | (a) Give characteristics of study participants (eg demographic, clinical, social) and information on exposures and potential confounders | NHANES cohorts: Page 6. Table 1  Hospital-based clinical cohort: Page 7. Table 2 |
|  |  | (b) Indicate number of participants with missing data for each variable of interest | Refer to "FIGURE 1" |
| Outcome data | 15* | Report numbers of outcome events or summary measures | Page 5. Results: 3.1 Baseline characteristics |
| Main results | 16 | (*a*) Give unadjusted estimates and, if applicable, confounder-adjusted estimates and their precision (eg, 95% confidence interval). Make clear which confounders were adjusted for and why they were included | NHANES cohorts: Page 8. Table 3  Hospital-based clinical cohort: Page 10. Table 4 |
|  |  | (*b*) Report category boundaries when continuous variables were categorized | Page 5. Results: 3.1 Baseline characteristics |
|  |  | (*c*) If relevant, consider translating estimates of relative risk into absolute risk for a meaningful time period | Not Applicable |
| Other analyses | 17 | Report other analyses done—eg analyses of subgroups and interactions, and sensitivity analyses | Page 7-8. Results: 3.5 Stratified analysis and subgroup analysis; 3.6 Analysis of the relationship between nutritional-inflammatory-related indices and DR severity in the hospital-based clinical cohort; 3.7 Sensitivity analysis |
| Discussion | | |  |
| Key results | 18 | Summarise key results with reference to study objectives | Page 9. Discussion: The second paragraph |
| Limitations | 19 | Discuss limitations of the study, taking into account sources of potential bias or imprecision. Discuss both direction and magnitude of any potential bias | Page 15. Discussion: The final paragraph |
| Interpretation | 20 | Give a cautious overall interpretation of results considering objectives, limitations, multiplicity of analyses, results from similar studies, and other relevant evidence | Page 9-15. Discussion: Comparing literature and exploring mechanisms |
| Generalisability | 21 | Discuss the generalisability (external validity) of the study results | Page 9. Discussion: The second paragraph |
| Other information | | |  |
| Funding | 22 | Give the source of funding and the role of the funders for the present study and, if applicable, for the original study on which the present article is based | Page 15. Funding |

*Give information separately for exposed and unexposed groups.

**Table S2 Unweighted logistic regression analysis of the association between nutritional-inflammatory-related indices and DR prevalence in the NHANES.**

| **Variable** | **Model 1** | | **Model 2** | | **Model 3** | | |
| --- | --- | --- | --- | --- | --- | --- | --- |
|  | **OR (95% CI)** | ***P* value** | **OR (95% CI)** | ***P* value** | **OR (95% CI)** | ***P* value** |  |
| **PNI**  Continuous  Q1  Q2  Q3  *P* for trend | 0.96 (0.95, 0.98)  Ref  0.67 (0.56, 0.81)  0.62 (0.50, 0.85) | **<0.001**  **<0.001**  **<0.001**  **<0.001** | 0.96 (0.95, 0.98)  Ref  0.66 (0.55, 0.80)  0.60 (0.49, 0.74) | **<0.001**  **<0.001**  **<0.001**  **<0.001** | 0.97 (0.96, 0.99)  Ref  0.73 (0.60, 0.89)  0.68 (0.55, 0.84) | **0.001**  **0.001**  **<0.001**  **<0.001** |  |
| **ALBI**  Continuous  Q1  Q2  Q3  *P* for trend | 1.97 (1.52, 2.54)  Ref  1.21 ( 0.98, 1.48)  1.54 ( 1.27, 1.87) | **<0.001**  0.073  **<0.001**  **<0.001** | 2.02 (1.56, 2.62)  Ref  1.24 (1.01, 1.53)  1.60 (1.31, 1.94) | **<0.001**  **0.040**  **<0.001**  **<0.001** | 1.50 (1.14, 1.97)  Ref  1.21 (0.98, 1.49)  1.35 (1.10, 1.66) | **0.004**  0.082  **0.005**  **<0.01** |  |
| **NPAR**  Continuous  Q1  Q2  Q3  *P* for trend | 1.08 (1.05, 1.11)  Ref  1.28 (1.05, 1.56)  1.55 (1.28, 1.88) | **<0.001**  **0.015**  **<0.001**  **<0.001** | 1.08 (1.05, 1.11)  Ref  1.32 (1.08, 1.62)  1.63 (1.34, 1.99) | **<0.001**  0.007  **<0.001**  **<0.001** | 1.05 (1.02, 1.08)  Ref  1.28 (1.04, 1.57)  1.38 (1.12, 1.70) | **<0.001**  **0.019**  **0.002**  **<0.01** |  |
| **GNRI**  Continuous  Q1  Q2  Q3  *P* for trend | 0.96 (0.94 0.97)  Ref  0.61 (0.51, 0.73)  0.58 (0.47, 0.72) | **<0.001**  **<0.001**  **<0.001**  **<0.001** | 0.95(0.94, 0.97)  Ref  0.59 (0.49, 0.71)  0.55 (0.44, 0.68) | **<0.001**  **<0.001**  **<0.001**  **<0.001** | 0.97 (0.95, 0.99)  Ref  0.67 (0.55, 0.81)  0.65 (0.52, 0.82) | **<0.001**  Ref  **<0.001**  **<0.001**  **<0.001** |  |

***Note:*** Model 1 was adjusted for none. Model 2 was adjusted for age, sex, race ,marital status, education level. Model 3 was adjusted for Model 2 variables plus smoking status, drinking status, BMI, HbA1c, eGFR, hypertension, hyperlipidemia.

***Abbreviations:*** *CI*, confidence interval; *OR*, odds ratio; *BMI*, body mass index; *eGFR*, estimated glomerular filtration rate; *HbA1c*, glycosylated hemoglobin A1c; *PNI*, prognostic nutritional index; *NPAR*, neutrophil percentage to albumin ratio; *ALBI*, albumin-bilirubin; *GNRI*, geriatric nutritional risk index.

**Table S3 The association between nutritional-inflammatory-related indices**

**and DR prevalence after outlier exclusion in the NHANES.**

| **Variable** | **Model 1** | | **Model 2** | | **Model 3** | | |
| --- | --- | --- | --- | --- | --- | --- | --- |
|  | **OR (95% CI)** | ***P* value** | **OR (95% CI)** | ***P* value** | **OR (95% CI)** | ***P* value** |  |
| **PNI**  Continuous  Q1  Q2  Q3  *P* for trend | 0.94 (0.91, 0.97)  Ref  0.53 (0.43, 0.80)  0.55 (0.40, 0.75) | **<0.001**  **0.001**  **<0.001**  **<0.001** | 0.94 (0.91, 0.97)  Ref  0.58 (0.42, 0.80)  0.54 (0.39, 0.74) | **<0.001**  **0.001**  **<0.001**  **<0.001** | 0.95 (0.91, 0.98)  Ref  0.58 (0.42, 0.82)  0.55 (0.39, 0.77) | **0.002**  **0.002**  **<0.001**  **<0.001** |  |
| **ALBI**  Continuous  Q1  Q2  Q3  *P* for trend | 2.28 ( 1.32, 3.94)  Ref  1.52 ( 1.11, 2.10)  1.64 ( 1.16, 2.32) | **0.003**  **0.010**  **0.005**  **<0.01** | 2.28 (1.34, 3.89)  Ref  1.55 (1.13, 2.14)  1.65 (1.18, 2.31) | **0.003**  **0.007**  **0.004**  **<0.01** | 1.95 (1.14, 3.34)  Ref  1.56 (1.13, 2.14)  1.53 (1.10, 2.14) | **0.015**  **0.007**  **0.012**  **<0.05** |  |
| **NPAR**  Continuous  Q1  Q2  Q3  *P* for trend | 1.06 (1.02, 1.11)  Ref  1.08 (0.82, 1.43  1.35 (1.02, 1.78) | **0.005**  0.572  **0.036**  **<0.05** | 1.07 (1.03, 1.12)  Ref  1.11 (0.83, 1.49)  1.41 (1.05, 1.88) | **0.003**  0.481  **0.023**  **<0.05** | 1.05 (1.00, 1.11)  Ref  1.12 (0.82, 1.52)  1.40 (1.01, 1.93) | **0.039**  0.484  **0.043**  **<0.05** |  |
| **GNRI**  Continuous  Q1  Q2  Q3  *P* for trend | 0.95 (0.92, 0.97)  Ref  0.70 (0.52, 0.95)  0.53 (0.37, 0.77) | **<0.001**  **0.021**  **<0.001**  **<0.001** | 0.95 (0.92, 0.97)  Ref  0.70 (0.51, 0.95)  0.52 (0.36, 0.73) | **<0.001**  **0.021**  **<0.001**  **<0.001** | 0.95 (0.92, 0.98)  Ref  0.72 (0.53, 0.98)  0.56 (0.39, 0.81) | **0.002**  Ref  **0.040**  **0.002**  **<0.01** |  |

***Note:*** Model 1 was adjusted for none. Model 2 was adjusted for age, sex, race ,marital status, education level. Model 3 was adjusted for Model 2 variables plus smoking status, drinking status, BMI, HbA1c, eGFR, hypertension, hyperlipidemia.

***Abbreviations:*** *CI*, confidence interval; *OR*, odds ratio; *BMI*, body mass index; *eGFR*, estimated glomerular filtration rate; *HbA1c*, glycosylated hemoglobin A1c; *PNI*, prognostic nutritional index; *NPAR*, neutrophil percentage to albumin ratio; *ALBI*, albumin-bilirubin; *GNRI*, geriatric nutritional risk index.

**Table S4 Association of nutritional-inflammatory-related indices with DR prevalence after additional adjustment for potential confounders in the NHANES.**

| **Variable** | **Model 1** | | **Model 2** | | **Model 3** | | |
| --- | --- | --- | --- | --- | --- | --- | --- |
|  | **OR (95% CI)** | ***P* value** | **OR (95% CI)** | ***P* value** | **OR (95% CI)** | ***P* value** |  |
| **PNI**  Continuous  Q1  Q2  Q3  *P* for trend | 0.95 (0.93, 0.97)  Ref  0.53 (0.40, 0.70)  0.54 (0.40, 0.71) | **<0.001**  **<0.001**  **<0.001**  **<0.001** | 0.95 (0.93, 0.97)  Ref  0.53 (0.40, 0.71)  0.52 (0.39, 0.71) | **<0.001**  **<0.001**  **<0.001**  **<0.001** | 0.95 (0.93, 0.98)  Ref  0.54 (0.40, 0.74)  0.55 (0.39, 0.78) | **<0.001**  **<0.001**  **<0.001**  **<0.001** |  |
| **ALBI**  Continuous  Q1  Q2  Q3  *P* for trend | 2.25 ( 1.55, 3.27)  Ref  1.50 ( 1.12, 2.01)  1.76 ( 1.30, 2.37) | **<0.001**  **0.007**  **<0.001**  **<0.001** | 2.18 (1.50, 3.15)  Ref  1.55 (1.15, 2.08)  1.76 (1.30, 2.37) | **<0.001**  **0.004**  **<0.001**  **<0.001** | 1.71 (1.14, 2.58)  Ref  1.51 (1.12, 2.03)  1.57 (1.14, 2.16) | **0.011**  **0.007**  **0.006**  **<0.01** |  |
| **NPAR**  Continuous  Q1  Q2  Q3  *P* for trend | 1.08 (1.04, 1.11)  Ref  1.12 (0.86, 1.45)  1.50 (1.18, 1.92) | **<0.001**  0.405  **0.001**  **<0.01** | 1.08 (1.04, 1.11)  Ref  1.15 (0.87, 1.52)  1.54 (1.19, 2.00) | **<0.001**  0.320  **0.001**  **<0.001** | 1.05 (1.01, 1.10)  Ref  1.11 (0.84, 1.48)  1.38 (1.02, 1.87) | **0.014**  0.458  **0.038**  **<0.05** |  |
| **GNRI**  Continuous  Q1  Q2  Q3  *P* for trend | 0.95 (0.93, 0.97)  Ref  0.63 (0.47, 0.84)  0.51 (0.38, 0.69) | **<0.001**  **0.002**  **<0.001**  **<0.001** | 0.95 (0.93, 0.97)  Ref  0.64 (0.48, 0.85)  0.50 (0.38, 0.67) | **<0.001**  **0.003**  **<0.001**  **<0.001** | 0.96 (0.94, 0.99)  Ref  0.69 (0.51, 0.91)  0.56 (0.41, 0.76) | **0.002**  Ref  **0.010**  **<0.001**  **<0.001** |  |

***Note:*** Model 1 was adjusted for none. Model 2 was adjusted for age, sex, race ,marital status, education level, PIR. Model 3 was adjusted for Model 2 variables plus smoking status, drinking status, BMI, HbA1c, eGFR, ALT, AST, RDW, hypertension, hyperlipidemia.

***Abbreviations:*** *CI*, confidence interval; *OR*, odds ratio; *BMI*, body mass index; *eGFR*, estimated glomerular filtration rate; *HbA1c*, glycosylated hemoglobin A1c; *PNI*, prognostic nutritional index; *NPAR*, neutrophil percentage to albumin ratio; *ALBI*, albumin-bilirubin; *GNRI*, geriatric nutritional risk index; *PIR*, family income-to-poverty ratio; *ALT*, alanine transaminase; *AST*, aspartate transaminase; *RDW*, red blood cell distribution width.

**Figure S1.** Spearman correlation heatmap among the four nutrition‑inflammation‑related indices in the NHANES cohort**.**
